# Alpha Frequency Dysrhythmia in Treatment-Resistant Schizophrenia: Associations with EEG Background Changes, Disorganized Symptoms, and Dissociation

**DOI:** 10.3390/biomedicines14071480

**Published:** 2026-06-30

**Authors:** Georgi Panov, Presyana Panova, Silvana Dyulgerova

**Affiliations:** 1 Psychiatric Clinic, University Hospital for Active Treatment “Prof. Dr. Stoyan Kirkovich”, Trakia University, 6000 Stara Zagora, Bulgaria; 2 Medical Faculty, University “Prof. Dr. Asen Zlatarov”, 8000 Burgas, Bulgaria; 3 Medical Faculty, Trakia University, 6000 Stara Zagora, Bulgaria

**Keywords:** alpha rhythm, dysrhythmia, treatment-resistant schizophrenia, EEG background changes, dissociation, disorganized symptoms, biomarker

## Abstract

**Background:** Treatment-resistant schizophrenia (TRS) affects approximately 20–30% of patients and is associated with significant disability. EEG abnormalities, particularly background slowing and disorganized alpha activity, have been reported in TRS, but the role of alpha rhythm instability—here termed alpha dysrhythmia—remains poorly understood. **Objective:** To compare the individual alpha frequency (IAF) between patients with TRS and those in clinical remission, to examine associations between alpha dysrhythmia and specific symptom domains (especially disorganization), and to investigate its relationship with EEG background changes. **Methods:** Eighty-nine patients with schizophrenia were included. Alpha dysrhythmia was defined as intraindividual variability of dominant alpha frequency exceeding 1 Hz across consecutive EEG epochs. Quantitative spectral analysis was performed using FFT on artifact-free 4–9 s epochs. Clinical assessment included PANSS (positive, negative, and disorganized subscales), the Dissociation scale, BPRS, Hamilton D/A, and the OCD scale. Group comparisons used the Mann–Whitney U test; correlations used Pearson and Spearman coefficients; and stepwise regression identified independent predictors. **Results:** Alpha dysrhythmia was present in 46.1% of patients. Significant negative correlations were found between dysrhythmia and therapeutic response. Significant positive correlations were found with PANSS disorganized symptoms and the Dissociation scale. The Mann–Whitney U test showed that the dysrhythmia group had higher mean ranks for EEG background factor (EEG BA), the Dissociation scale, and PANSS disorganized symptoms. Stepwise regression identified EEG BA and the Dissociation scale as independent predictors. **Conclusions:** Alpha dysrhythmia is frequent in TRS patients and is specifically associated with poorer therapeutic response, disorganized symptoms, and dissociation. EEG BA (reflecting background changes) may serve as a neurophysiological biomarker for identifying patients at risk for treatment resistance.

## 1. Introduction

Schizophrenia is a severe, chronic psychiatric disorder with a lifetime prevalence of approximately 1% of the general population, affecting over 20 million people worldwide and characterized by significant cognitive impairments and poor social and occupational outcomes [1,2,3]. The clinical picture includes three main symptom dimensions: positive symptoms (delusions, hallucinations), negative symptoms (apathy, avolition, reduced emotional expression), and disorganized symptoms (disorganized thinking and behavior) [4,5,6,7]. Cognitive symptoms often appear significantly earlier than psychotic manifestations and represent a major obstacle to functional recovery [8,9].

Contemporary neuroscience models place schizophrenia in the context of predictive coding theory [10,11]. According to this theory, the brain continuously generates predictions about external stimuli and updates its internal models based on prediction errors [12,13]. In schizophrenia, there is an increased precision of low-level prediction errors, which leads to aberrant salience attribution to irrelevant stimuli and subsequent distortion of reality [14,15,16]. Evidence for this comes from studies of auditory mismatch negativity (MMN), which is considered an electrophysiological marker of prediction errors and is consistently reduced in patients with schizophrenia and individuals at clinical high risk for psychosis [17,18,19,20].

At the molecular level, the pathogenesis of schizophrenia is closely related to two main neurotransmitter imbalances. The first is hyperdopaminergia in mesolimbic pathways and hypodopaminergia in mesocortical areas [21,22]. Dopamine plays a critical role as a modulator of the signal-to-noise ratio in cortical microcircuits, influencing the excitability of pyramidal neurons and recurrent inhibition via D1 and D2 receptors [23,24]. In schizophrenia, the abnormal ratio between D1 and D2 activation leads to a disrupted cortical signal-to-noise ratio, affecting the stability of neuronal representations of external and internal stimuli [25,26]. This enables prioritized processing of irrelevant stimuli, which underlies delusion formation as a form of reality distortion [27]. 

The second key mechanism is NMDA receptor hypofunction [28]. Since the early 2000s, substantial evidence has accumulated that subanesthetic doses of NMDA antagonists (e.g., ketamine) reproduce in healthy individuals the cognitive deficits, negative symptoms, and brain functional abnormalities characteristic of schizophrenia [29,30]. Postmortem and genetic studies identify multiple schizophrenia-related abnormalities that prevent activation of the glycine modulatory site of the NMDA receptor [31,32]. This leads to reduced reactivity to external stimuli, which clinically manifests as negative symptoms—lack of expression, apathy, avolition, and general emotional blunting [33,34,35]. The imbalance between dopaminergic and glutamatergic systems, together with the involvement of molecules such as neuregulin/ErbB4, leads to regression of dendritic spines, further deepening neuronal dysfunction [36,37].

The present study is part of a larger, ongoing research project investigating clinical and neurophysiological markers in schizophrenia, using the same core cohort of 89 patients. Parts of this project have been previously published, focusing on various clinical dimensions including obsessive-compulsive symptoms [38], dissociative features [39], lateralization [40], gender differences [41], depressive symptoms [42], the effect of the first antipsychotic drug [43], education level and sex differences [44], anthropometric criteria [45], the role of disorganized symptoms and duration of untreated psychosis [46], and paroxysmal EEG activity in relation to treatment resistance [47]. The current analysis extends this work by examining alpha rhythm dysrhythmia in relation to treatment resistance and EEG features.

An additional key molecular factor that integrates glutamatergic and dopaminergic dysregulation is the imbalance in the kynurenine pathway of tryptophan metabolism. Kynurenic acid (KYNA) is an endogenous metabolite that acts as an antagonist at both NMDA receptors (at the glycine site) and α7 nicotinic acetylcholine receptors (α7nAChR) [48,49,50]. In schizophrenia, elevated KYNA levels are consistently found in postmortem prefrontal cortex and cerebrospinal fluid, leading to functional hypoglutamatergia and further deepening of cognitive deficits [51]. The metabolic flux toward KYNA is determined by the activity of the enzymes kynurenine-3-monooxygenase (KMO) and kynurenine aminotransferases (KATs). In schizophrenia, the expression and activity of the microglial enzyme KMO are significantly reduced, redirecting L-kynurenine metabolism toward astroglial synthesis of KYNA instead of neurotoxic 3-hydroxykynurenine [52]. This “shift in direction” of metabolism leads to KYNA overproduction.

Of particular relevance to the electrophysiological findings in schizophrenia is the ability of KYNA to influence neuronal oscillations. By blocking α7nAChR and NMDA receptors, KYNA reduces the excitability of GABAergic interneurons in the hippocampus and prefrontal cortex [49,50,53,54]. These interneurons are essential for generating alpha and gamma oscillations, which underlie sensory gating—the mechanism for filtering irrelevant sensory information [55,56]. Elevated KYNA levels lead to reduced precision of neuronal synchronization and to dysrhythmia [57,58].

In patients with schizophrenia, elevated serum KYNA levels are significantly associated with impaired P50 sensory gating—an electrophysiological marker reflecting the ability to suppress response to a repeated stimulus [59,60]. This deficit correlates with the severity of disorganized symptoms and with abnormalities in the alpha frequency range of the EEG [59,61]. Therefore, the dysrhythmia in the linear frequency of the alpha rhythm may be directly mediated by elevated KYNA levels and subsequent GABAergic dysfunction [62,63].

The electroencephalographic (EEG) alpha rhythm (8–13 Hz) is the dominant rhythm in the resting state with eyes closed and reflects the fundamental processes of neuronal excitability and synchronization in thalamocortical circuits [64,65]. The alpha rhythm plays an active role in inhibiting irrelevant sensory information and maintaining internal representations [66,67]. It participates in “filling in” missing sensory data with predictions generated by the brain’s internal models, which is key to building an accurate model of reality [68]. Importantly, the linear frequency of the alpha rhythm (Individual Alpha Frequency—IAF), not just its power, is critical for the temporal resolution of sensory processing and is altered in patients with schizophrenia [69,70,71].

Approximately 20–30% of patients with schizophrenia develop treatment-resistant schizophrenia (TRS) [3,72]. These patients show distinct EEG characteristics that distinguish them from patients in remission. First, they have a significantly higher prevalence of interictal epileptiform discharges (IEDs)—sharp waves, spikes, and spike-wave complexes—occurring in up to 43.6% of TRS patients compared to only 20% in those in remission [66,73,74]. These paroxysmal changes are predominantly localized in temporal and frontal regions, often showing left hemispheric dominance [66]. Second, TRS patients demonstrate significantly more pronounced slowing of the background EEG activity, with a predominance of theta and delta power and reduced alpha activity, reflecting diffuse cortical dysfunction and impaired thalamocortical regulation [47,75,76]. In the TRS group, 69.2% of patients fall into the category of moderate to severe background abnormalities, whereas in the remission group 86.0% show normal or minimally expressed changes [66]. These EEG findings are independent predictors of therapeutic resistance [47,77,78].

Previous research from our group has demonstrated that disorganized symptoms are closely associated with treatment resistance in schizophrenia [46]. Given that disorganized symptoms have been linked to sensory gating deficits and disturbances in predictive coding, we specifically hypothesized that alpha dysrhythmia would be associated with this symptom dimension.

Based on the foregoing, the aims of the present study were:

1 To compare the linear frequency of the alpha rhythm (IAF) between patients with treatment-resistant schizophrenia (TRS) and those in clinical remission.
2 To examine the relationship between dysrhythmia in IAF and specific symptom domains.
3 To investigate the association between IAF abnormalities and EEG background changes and paroxysmal events.

## 2. Materials and Methods

### 2.1. Participants

This study included patients diagnosed with schizophrenia according to DSM-5 criteria [79,80], divided into two groups: (1) patients with treatment-resistant schizophrenia (TRS), defined according to the TRRIP consensus criteria (lack of adequate response to at least two different antipsychotics at sufficient dose and duration) [3,72,81], and (2) patients in clinical remission, defined according to the consensus criteria of Andreasen et al. [82].

Exclusion criteria were neurological disorders, traumatic brain injury, substance abuse during the last 6 months, electroconvulsive therapy during the last 3 months, intellectual disability, active substance abuse disorder, documented organic brain damage, concomitant progressive neurological or severe somatic illness, pronounced personality change (assessed via DSM-5/ICD-10 criteria), a score below 25 on the Mini-Mental State Examination (MMSE), and pregnancy or lactation. All participants gave written informed consent, and the protocol was approved by the local ethics committee.

### 2.2. Clinical Assessment

The severity of symptoms was assessed using the Positive and Negative Syndrome Scale (PANSS) [83], with separate analysis of the three factor solutions: positive symptoms, negative symptoms, and disorganized symptoms (cognitive/disorganization factor) [5,6]. Additional clinical assessments included the Dissociation Scale, the Brief Psychiatric Rating Scale (BPRS) [84], the Hamilton Depression Rating Scale (Hamilton D), the Hamilton Anxiety Rating Scale (Hamilton A), and the OCD scale.

### 2.3. EEG Recording and Analysis

EEG activity was recorded in a waking state with eyes closed at rest, with participants instructed to remain relaxed and avoid movements. The recording was performed with 19 electrodes positioned according to the international 10–20 system, with a linked-mastoid reference configuration and impedance kept below 5 kΩ. The signal was amplified, filtered with a band-pass filter 0.5–70 Hz, and digitized at a sampling frequency of 256 Hz using the Mitsar-EEG system (Mitsar Ltd., St. Petersburg, Russia). The standard recording protocol included consecutive periods of rest with eyes closed and eyes open, hyperventilation (3–5 min) in the absence of contraindications, and intermittent photic stimulation. The minimum duration of interpretable brain activity recording was 20 min, in accordance with international standards [85,86]. Spectral analysis was performed exclusively on the eyes-closed resting segments, as alpha rhythm is most robustly expressed in this state.

Quantitative spectral analysis was performed using the built-in Fast Fourier Transform (FFT) module of the Win-EEG software (version 2.138.111; Mitsar Ltd.). The software applies Welch’s averaged periodogram method with the following default parameters: Hamming window, 50% overlap between consecutive segments, and a frequency resolution of 0.5 Hz. The power spectral density (PSD) was computed for each visually inspected, artifact-free epoch (duration: 4–9 s). Epoch durations varied between 4 and 9 s depending on the length of artifact-free segments available for each patient. Only segments free of ocular, muscular, movement, and technical artifacts were included in the analysis.

The dominant alpha frequency (Individual Alpha Frequency, IAF) for each epoch was determined as the peak within the 8–13 Hz range of the PSD, averaged across all 19 channels, using the spectral analysis module of Win-EEG. Alpha dysrhythmia was operationally defined as intraindividual variability in IAF exceeding 1 Hz across consecutive artifact-free EEG epochs [66]. Short EEG epochs (4–9 s) have been widely applied in quantitative EEG research to improve spectral reliability and reduce contamination from state transitions and vigilance fluctuations [87,88,89,90]. A minimum of 5 epochs per patient was required for inclusion in the analysis, consistent with previous studies demonstrating that IAF can be reliably estimated from a limited number of short EEG epochs [91,92]. In cases where data quality was uncertain, additional epochs were included to refine the IAF estimation. All 89 patients in the final sample met this inclusion criterion.

The following EEG parameters were defined for the analysis:

- EEG BA (Background Activity): A composite factor reflecting the degree of background slowing, assessed through the ratio of theta (4–8 Hz) and delta (0.5–4 Hz) power to alpha (8–13 Hz) power, and the degree of alpha rhythm disorganization. Scores ranged from 1 (normal) to 4 (severe abnormality), based on visual and computational assessment.
- EEG FocA (Focal Activity): Presence of focal slowing or sharp waves in specific brain regions.
- EEG PA (Paroxysmal Activity): Presence of epileptiform discharges (spikes, sharp waves, spike-wave complexes), assessed visually.

Spectral analysis was performed on all 19 recording channels using the linked-ears reference configuration ((A1 + A2)/2), which is the standard clinical reference for this system [2,9]. Importantly, while this reference may affect signal amplitude in temporal regions, our primary measure—the Individual Alpha Frequency (IAF)—is based on the peak frequency within the power spectrum, which is robust to reference choice, as the subtraction of reference signals does not alter the frequency components of the EEG [93]. All reported results are therefore valid for the linked-ears reference configuration.

Alpha oscillations have been shown to reflect dynamic rather than fixed-frequency processes, with intrinsic temporal fluctuations in peak frequency over time [64,65,87,91,92,94]. Therefore, variability across epochs was considered a meaningful neurophysiological feature rather than measurement noise [66].

### 2.4. Statistical Analysis

Group comparisons (treatment-resistant schizophrenia vs. patients in remission) were performed using the Mann–Whitney U test, as the data did not meet normality assumptions [95]. Correlations between alpha dysrhythmia indices and clinical variables were assessed using Pearson and Spearman correlation coefficients. Multivariate analysis was performed using stepwise multiple linear regression to identify independent predictors of alpha dysrhythmia. Statistical significance was set at *p* < 0.05 (two-tailed). Statistical analysis was performed using SPSS (version 26.0) [95] or JASP [96].

### 2.5. Ethical Considerations

The study was conducted in accordance with the ethical principles of the Declaration of Helsinki. The study protocol was reviewed and approved by the Ethical Committee of the University Hospital “Prof. Dr. Stoyan Kirkovich” in Stara Zagora (Protocol Code: TR3--02-242, Date: 30 December 2021). All participants, or their legal guardians, provided written informed consent after receiving a detailed explanation of the study procedures, their safety, and the confidentiality of the data obtained.

## 3. Results

### 3.1. Demographic and Clinical Characteristics of the Study Group

The demographic and clinical characteristics of the patients studied are presented in detail in Table 1 (see also Panov et al., 2024 [46,47]). In brief, the analysis included 89 patients with schizophrenia. Of these, 48 patients (53.9%) were classified with normal alpha rhythm and 41 patients (46.1%) with alpha dysrhythmia. As shown in Table 1, the two groups were well-matched on key demographic parameters. There were no significant differences in age, height, weight, or Body Mass Index (BMI) between patients with TRS and those in remission (*p* > 0.05 for all). However, the age of onset was significantly earlier in the TRS group (23.33 ± 7.31 years) compared to the remission group (27.52 ± 8.45 years; *p* = 0.018). Consequently, the duration of schizophrenia was significantly longer in the TRS group (13.69 ± 11.45 years) than in the remission group (9.14 ± 6.98 years; *p* = 0.016).

### 3.2. Group Comparisons—Mann-Whitney U Test

To identify variables that best discriminate between the two groups (normal alpha rhythm vs. alpha dysrhythmia), a non-parametric Mann–Whitney U test was applied. Results are presented in Table 2.

As shown in Table 2, the dysrhythmia group had significantly higher mean ranks for all EEG parameters (EEG BA, EEG FocA, EEG PA) and for the Dissociation scale. The largest effect size was observed for EEG BA (r = 0.57, large effect), followed by EEG FoA (r = 0.38, medium effect) and EEG PA (r = 0.32, medium effect). The Dissociation scale showed a medium effect (r = 0.31), while PANSS disorganized symptoms showed a small to medium effect (r = 0.27).

The dysrhythmia group had significantly higher mean ranks for all EEG parameters: EEG BA (mean rank 60.37 vs. 31.88, U = 354.0, Z = −5.420, *p* < 0.001), EEG FocA (mean rank 54.05 vs. 37.27, U = 613.0, Z = −3.628, *p* < 0.001), and EEG PA (mean rank 52.12 vs. 38.92, U = 692.0, Z = −3.018, *p* = 0.003). The Dissociation scale also showed significantly higher ranks in the dysrhythmia group (mean rank 53.67 vs. 37.59, U = 628.5, Z = −2.927, *p* = 0.003). PANSS disorganized symptoms were elevated in the dysrhythmia group (mean rank 52.52 vs. 38.57, U = 675.5, Z = −2.544, *p* = 0.011), as were PANSS positive symptoms (mean rank 51.40 vs. 39.53, U = 721.5, Z = −2.168, *p* = 0.030).

Effect sizes (r) were calculated as Z/√N (N = 89, √N = 9.43). The largest effect size was observed for EEG BA (r = 0.57, large effect), followed by EEG FocA (r = 0.38, medium effect), EEG PA (r = 0.32, medium effect), the Dissociation scale (r = 0.31, medium effect), and PANSS disorganized symptoms (r = 0.27, small-to-medium effect).

After applying Bonferroni correction for multiple comparisons (α = 0.05/21 = 0.0024-based on the total number of comparisons performed across all analyses), EEG BA, EEG FocA, EEG PA, and the Dissociation scale remained statistically significant. PANSS disorganized symptoms (*p* = 0.011) and PANSS positive symptoms (*p* = 0.030) were significant at the uncorrected level but did not survive correction.

Importantly, no significant differences were observed between groups for PANSS negative symptoms (*p* = 0.197), BPRS (*p* = 0.520), neurocognitive variables (fixation, reproduction, retention; all *p* > 0.05), depressive symptoms (Hamilton D, *p* = 0.885; Hamilton A, *p* = 0.670), or the OCD scale (*p* = 0.859). These findings support the specificity of the association of alpha dysrhythmia with EEG background changes, dissociation, and disorganized symptoms.

### 3.3. Regression Analysis—Independent Predictors of Alpha Dysrhythmia

To identify independent predictors of alpha dysrhythmia, a stepwise multiple linear regression analysis was performed. The dependent variable was alpha dysrhythmia (1 = normal, 2 = dysrhythmia). All EEG and clinical variables were included in the model (n = 89). Results are presented in Table 3.

A one-standard-deviation increase in EEG BA was associated with a 0.353 standard deviation increase in the probability of alpha dysrhythmia (Beta = 0.353, moderate to large effect). Similarly, a one-unit increase in the Dissociation scale was associated with a 0.007-unit increase in alpha dysrhythmia (Beta = 0.296, moderate effect). The two predictors together explained 23.6% of the variance (R^2^ = 0.236) in alpha dysrhythmia.

After the inclusion of EEG BA and the Dissociation scale, no other variable reached the inclusion threshold (*p*-in > 0.05), including EEG PA (Beta In = 0.001, *p* = 0.995), PANSS disorganized (Beta In = 0.060, *p* = 0.620), and all other clinical variables. This confirms that EEG BA and the Dissociation scale are the strongest independent predictors of alpha dysrhythmia.

**Figure 1:**
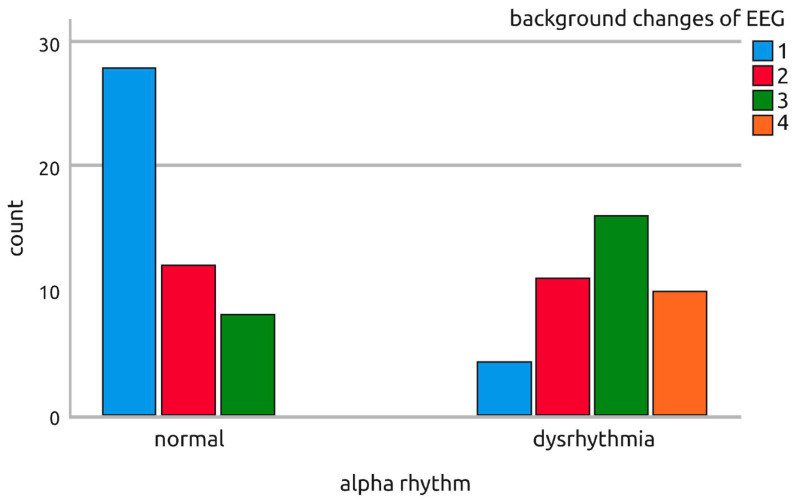
Distribution of alpha dysrhythmia and EEG background severity.

To further investigate the relationship between alpha dysrhythmia and the severity of EEG background changes (as measured by the EEG BA score), we performed a cross-tabulation analysis (Table 4; Figure 1). The distribution of patients with normal alpha rhythm and alpha dysrhythmia across the four EEG BA severity levels (1—normal to 4—severe) was significantly different (χ^2^ (3) = 33.63, *p* < 0.001). Notably, while all patients with the most severe background changes (EEG BA = 4) exhibited alpha dysrhythmia, the highest concentration of dysrhythmia was observed in the mild and moderate severity categories (EEG BA = 2 and 3). Conversely, the majority of patients with a normal EEG background (EEG BA = 1) had a normal alpha rhythm (28 out of 32, 87.5%). These findings suggest that alpha dysrhythmia is not merely an epiphenomenon of severe background slowing but emerges as a distinct feature even at mild-to-moderate levels of EEG background dysregulation.

## 4. Discussion

The present study demonstrates that alpha dysrhythmia—instability in the dominant alpha frequency—is significantly more prevalent in patients with treatment-resistant schizophrenia (46.1%) and is specifically associated with EEG background changes, dissociation, and disorganized symptoms.

The dysrhythmia group showed markedly higher EEG BA values (mean rank 60.37 vs. 31.88, *p* < 0.001, large effect size r = 0.57), indicating that background EEG slowing, theta/delta excess, and alpha disorganization are core features of TRS. This is consistent with previous studies linking diffuse cortical dysfunction and impaired thalamocortical regulation to poor antipsychotic response [66,75,76,95,97]. The negative correlation between alpha dysrhythmia and therapeutic response (r = −0.320, *p* = 0.002) further supports the notion that an unstable alpha rhythm reflects reduced temporal stability of thalamocortical coupling mechanisms [98,99]. Patients with such instability may have neuronal networks that are less susceptible to modulation by dopamine antagonists, potentially explaining their poorer response to first-line antipsychotics [21,27].

A key contribution of this study is the demonstration that alpha dysrhythmia is specifically associated with disorganized symptoms (PANSS disorganized, *p* = 0.011, r = 0.27) and dissociation (*p* = 0.003, r = 0.31), but not with depressive symptoms (Hamilton D/A), general psychopathology (BPRS), or obsessive-compulsive symptoms (OCD scale). Disorganized symptoms in schizophrenia are linked to functional dysregulation of the prefrontal cortex and its connections with temporal regions [5,6]. Dissociation—a sense of detachment from one’s own body, environment, or thought stream—is characterized by disruption of integration among perception, memory, identity, and awareness [66,67]. The alpha rhythm plays an active role in inhibiting irrelevant sensory information and maintaining internal representations [66,67]. Disruption of this mechanism (expressed as dysrhythmia) would logically lead to dissociative experiences. The specificity of this association is supported by the lack of significant differences in depressive symptoms between groups.

The stepwise regression analysis identified EEG BA as the strongest independent predictor of alpha dysrhythmia (moderate to large effect size). The addition of the Dissociation scale modestly improved the model, but the two predictors together explained only 23.6% of the variance, suggesting that other factors—likely neurochemical and inflammatory—contribute to the phenomenon.

The relationship between alpha dysrhythmia and the severity of background EEG changes warrants further comment. Our cross-tabulation analysis (Table 4) revealed that while all patients with the most severe background abnormalities (EEG BA = 4) presented with alpha dysrhythmia, the largest number of dysrhythmia cases accumulated in the mild and moderate severity groups (EEG BA = 2 and 3). Importantly, a substantial proportion of patients with a normal EEG background (EEG BA = 1) maintained a normal alpha rhythm (87.5%). This pattern suggests that once a certain level of background EEG dysregulation is reached (beyond normal), the tendency for the dominant alpha frequency to become unstable (i.e., dysrhythmia) significantly increases. The emergence of dysrhythmia already at the mild and moderate stages of background slowing, and not exclusively in the severe category, indicates that alpha dysrhythmia is not simply a proxy for the global severity of the EEG abnormality. Instead, our data support the conceptualization of alpha dysrhythmia as a semi-independent neurophysiological signature, potentially reflecting a specific dysfunction of thalamocortical circuits and GABA-ergic interneuron stability. This interpretation aligns with our regression model, where EEG BA and the Dissociation scale emerged as independent predictors, and further reinforces the potential of alpha dysrhythmia as a sensitive, early biomarker for network instability in treatment-resistant schizophrenia.

This finding is consistent with the kynurenine pathway hypothesis of schizophrenia [41,42,51,97,98]. Elevated kynurenic acid (KYNA) levels reduce the excitability of GABAergic interneurons [49,50,53,54]—the very neurons that are critical for generating alpha oscillations [55,56]. KYNA-induced GABAergic dysfunction would lead to reduced precision of neuronal synchronization and, consequently, to alpha dysrhythmia. This aligns with the integrative model: inflammation → KYNA ↑ → GABAergic dysfunction → alpha dysrhythmia → disorganized symptoms.

Our findings align with contemporary models of schizophrenia as a disorder of large-scale brain network dysregulation [99,100]. Recent work by Battaglia and colleagues has emphasized how excitatory-inhibitory imbalance can propagate through cortical networks, leading to widespread dysregulation of neural oscillations and cognitive deficits [101,102].

### 4.1. Clinical Implications

Our findings have several potential clinical applications: (1) Diagnostic biomarker—EEG BA effectively discriminates between patients with and without alpha dysrhythmia; (2) Predictor of therapeutic response—patients with significant alpha dysrhythmia may require alternative therapeutic strategies (e.g., clozapine); (3) Neurophysiological stratification—the identification of EEG background changes and dissociation as independent predictors suggests different neurophysiological subtypes of treatment resistance.

### 4.2. Limitations and Future Directions

Several limitations of this study should be acknowledged. First, the cross-sectional design precludes causal inferences regarding the relationship between alpha dysrhythmia and treatment resistance. Second, the sample size (n = 89), while adequate for the main analyses, may limit the generalizability of our findings to broader populations with schizophrenia. Third, we did not directly measure kynurenic acid (KYNA) or other inflammatory markers; therefore, the proposed link between KYNA, GABAergic dysfunction, and alpha dysrhythmia remains speculative and requires direct validation in future studies. Fourth, medication effects were not systematically controlled, although all patients were on stable antipsychotic regimens at the time of EEG recording. Fifth, the use of linked-ears reference configuration ((A1 + A2)/2) may introduce some distortion of signal amplitude in temporal regions due to volume conduction from nearby cortical sources. However, since our primary measure was the Individual Alpha Frequency (IAF)—a frequency-based metric rather than an amplitude-based one—this limitation is unlikely to have affected our main findings. Nevertheless, future studies could validate our results using alternative reference montages such as average reference or REST (Reference Electrode Standardization Technique). Sixth, our spectral analysis was performed using the built-in FFT module of Win-EEG with standard clinical parameters. While this approach is clinically validated, future studies may benefit from exporting raw data in ASCII format for more detailed post-processing, such as applying alternative spectral estimation methods or different frequency resolution parameters, to further validate the robustness of alpha dysrhythmia as a biomarker.

Future research should include prospective longitudinal studies, direct measurement of KYNA and inflammatory markers, interventional studies with GABAergic modulators, neuroimaging studies, and multi-center replication.

## 5. Conclusions

This study demonstrates that alpha dysrhythmia is a common phenomenon in patients with treatment-resistant schizophrenia (46.1%) and is specifically associated with EEG background changes, dissociation, and disorganized symptoms. EEG BA (reflecting background slowing, theta/delta power, and alpha disorganization) and the Dissociation scale emerged as the strongest independent predictors. The findings support an integrative model linking inflammation, kynurenic acid, GABAergic dysfunction, and alpha dysrhythmia to disorganized symptoms. From a clinical perspective, dysrhythmia may serve as a simple, accessible neurophysiological biomarker for identifying patients at risk for treatment resistance.

## Figures and Tables

**Table 1:** Demographic and clinical characteristics of the study sample (N = 89).

Characteristic	TRS (n = 39) M ± SD	Remission (n = 50) M ± SD	Total (N = 89) M ± SD	*p*-Value
Age (years)	36.82 ± 10.79	36.84 ± 10.26	36.83 ± 10.48	0.994
Age of Onset (years)	23.33 ± 7.31	27.52 ± 8.45	25.68 ± 8.12	0.018
Duration of Illness (years)	13.69 ± 11.45	9.14 ± 6.98	11.14 ± 9.22	0.016
Height (sm)	169.79 ± 8.56	166.84 ± 7.45	168.13 ± 8.09	0.073
Weight (kg)	75.00 ± 15.04	74.66 ± 16.24	74.81 ± 15.70	0.919
BMI (kg/m^2^)	26.39 ± 4.86	26.88 ± 5.61	26.66 ± 5.27	0.667

M—mean; SD—standard deviation; BMI—Body Mass Index.

**Table 2:** Mann–Whitney test for assessment of variables connected with the differences between the two groups.

Variable	Mean Rank (Normal, n = 48)	Mean Rank (Dysrhythmia, n = 41)	U	Z	*p*	Effect Size (r)
EEG BA	31.88	60.37	354.0	−5.420	<0.001	0.574
EEG PA	38.92	52.12	692.0	−3.018	0.003	0.319
EEG FocA	37.27	54.05	613.0	−3.628	<0.001	0.384
Dissociation scale	37.59	53.67	628.5	−2.927	0.003	0.310
PANSS disorganized	38.57	52.52	675.5	−2.544	0.011	0.269
PANSS positive	39.53	51.40	721.5	−2.168	0.030	0.229
PANSS negative	41.74	48.82	827.5	−1.291	0.197	0.137
BPRS	43.56	46.66	913.0	−0.643	0.520	0.068
Fixation	48.23	41.22	829.0	−1.280	0.200	0.135
Reproduction	47.38	42.54	838.0	−1.008	0.313	0.107
Retention	46.83	43.23	868.0	−0.728	0.467	0.077
Hamilton D	44.64	45.43	966.5	−0.145	0.885	0.015
Hamilton A	46.07	43.74	932.5	−0.426	0.670	0.045
OCD scale	45.42	44.56	955.0	−0.177	0.859	0.019

EEG BA—background activity, EEG PA—paroxysmal activity, EEG FocA—focal activity. Note: EEG BA—EEG factor analysis (reflects background slowing, theta/delta power, and alpha disorganization); EEG FocA—focal activity; EEG PA—paroxysmal activity; PANSS—Positive and Negative Syndrome Scale. According to Cohen’s conventions: r ≥ 0.5 = large effect; 0.3 ≤ r < 0.5 = medium effect; 0.1 ≤ r < 0.3 = small effect. Bonferroni-corrected threshold for multiple comparisons: α = 0.0031 (0.05/16).

**Table 3:** Stepwise multiple linear regression analysis for predictors of alpha dysrhythmia.

Model	Variable	B (Unstd.)	Std. Error	Beta (Std.)	t	*p*	95% CI Lower	95% CI Upper
1	(Constant)	0.862	0.100	–	8.598	<0.001	0.663	1.061
	EEG BA	0.280	0.042	0.579	6.632	<0.001	0.196	0.364
2	(Constant)	0.769	0.149	–	5.164	<0.001	0.473	1.065
	EEG BA	0.362	0.097	0.353	3.717	<0.001	0.169	0.555
	Dissociation scale	0.007	0.002	0.296	3.114	0.003	0.003	0.011

Model 1 (single factor): The only variable entered was EEG BA. R = 0.579, R^2^ = 0.336, Adjusted R^2^ = 0.328, F (1,87) = 43.98, *p* < 0.001. Model 2 (two factors): Variables entered: EEG BA and Dissociation scale. R = 0.486, R^2^ = 0.236, Adjusted R^2^ = 0.218, F (2,86) = 13.27, *p* < 0.001. Coefficients for Model 2: (Constant): B = 0.769 (SE = 0.149), t = 5.164, *p* < 0.001, 95% CI [0.473, 1.065]. EEG BA: B = 0.362 (SE = 0.097), Beta = 0.353, t = 3.717, *p* < 0.001, 95% CI [0.169, 0.555]. Dissociation scale: B = 0.007 (SE = 0.002), Beta = 0.296, t = 3.114, *p* = 0.003, 95% CI [0.003, 0.011].

**Table 4:** Cross-tabulation of alpha dysrhythmia and EEG background severity (EEG BA scores).

Alpha Rhythm Dysrhythmia\EEG BA	1 (Normal)	2 (Mild)	3 (Moderate)	4 (Severe)	Total
Normal	28	12	8	0	48
Dysrhythmia	4	11	16	10	41
Total	32	23	24	10	89

## Data Availability

The raw data supporting the conclusions of this article will be made available by the authors upon reasonable request. The data are not publicly available due to privacy restrictions.

## References

[1] GBD 2019 Mental Disorders Collaborators (2022). Global, regional, and national burden of 12 mental disorders in 204 countries and territories, 1990–2019: A systematic analysis for the Global Burden of Disease Study 2019. Lancet Psychiatry.

[2] Kahn R.S., Sommer I.E., Murray R.M., Meyer-Lindenberg A., Weinberger D.R., Cannon T.D., O’Donovan M., Correll C.U., Kane J.M., van Os J. (2015). Schizophrenia. Nat. Rev. Dis. Prim..

[3] Howes O.D., McCutcheon R., Agid O., de Bartolomeis A., van Beveren N.J., Birnbaum M.L., Bloomfield M.A., Bressan R.A., Buchanan R.W., Carpenter W.T. (2017). Treatment-Resistant Schizophrenia: Treatment Response and Resistance in Psychosis (TRRIP) Working Group Consensus Guidelines on Diagnosis and Terminology. Am. J. Psychiatry.

[4] Andreasen N.C. (1995). Symptoms, signs, and diagnosis of schizophrenia. Lancet.

[5] Liddle P.F. (1987). The symptoms of chronic schizophrenia. Br. J. Psychiatry.

[6] Marder S.R., Galderisi S. (2017). The current conceptualization of negative symptoms in schizophrenia. World Psychiatry.

[7] Correll C.U., Schooler N.R. (2020). Negative Symptoms in Schizophrenia: A Review and Clinical Guide for Recognition, Assessment, and Treatment. Neuropsychiatr. Dis. Treat..

[8] Green M.F., Horan W.P., Lee J. (2015). Social cognition in schizophrenia. Nat. Rev. Neurosci..

[9] Kahn R.S., Keefe R.S. (2013). Schizophrenia is a cognitive illness: Time for a change in focus. JAMA Psychiatry.

[10] Friston K.J. (2005). Hallucinations and perceptual inference. Behav. Brain Sci..

[11] Corlett P.R., Horga G., Fletcher P.C., Alderson-Day B., Schmack K., Powers A.R. (2019). Hallucinations and Strong Priors. Trends Cogn. Sci..

[12] Rao R.P.N., Ballard D.H. (1999). Predictive coding in the visual cortex: A functional interpretation of some extra-classical receptive-field effects. Nat. Neurosci..

[13] Heekeren H.R., Marrett S., Ungerleider L.G. (2008). The neural systems that mediate human perceptual decision making. Nat. Rev. Neurosci..

[14] Adams R.A., Stephan K.E., Brown H.R., Frith C.D., Friston K.J. (2013). The computational anatomy of psychosis. Front. Psychiatry.

[15] Sterzer P., Adams R.A., Fletcher P., Frith C., Lawrie S.M., Muckli L., Petrovic P., Uhlhaas P., Voss M., Corlett P.R. (2018). The Predictive Coding Account of Psychosis. Biol. Psychiatry.

[16] Heinz A., Schlagenhauf F. (2010). Dopaminergic dysfunction in schizophrenia: Salience attribution revisited. Schizophr. Bull..

[17] Umbricht D., Krljes S. (2005). Mismatch negativity in schizophrenia: A meta-analysis. Schizophr. Res..

[18] Näätänen R., Todd J., Schall U. (2016). Mismatch negativity (MMN) as biomarker predicting psychosis in clinically at-risk individuals. Biol. Psychol..

[19] Light G.A., Näätänen R. (2013). Mismatch negativity is a breakthrough biomarker for understanding and treating psychotic disorders. Proc. Natl. Acad. Sci. USA.

[20] Anita N.Z., Zebarth J., Chan B., Wu C.-Y., Syed T., Shahrul D., Nguyen M.M., Pakosh M., Herrmann N., Lanctôt K.L. (2022). P93. Inflammatory Markers in Type 2 Diabetes and Cognitive Impairment: A Systematic Review and Meta-Analysis. Biol. Psychiatry.

[21] Howes O.D., Kapur S. (2009). The dopamine hypothesis of schizophrenia: Version III—The final common pathway. Schizophr. Bull..

[22] Weinberger D.R. (1987). Implications of normal brain development for the pathogenesis of schizophrenia. Arch. Gen. Psychiatry.

[23] Winterer G., Weinberger D.R. (2004). Genes, dopamine and cortical signal-to-noise ratio in schizophrenia. Trends Neurosci..

[24] Durstewitz D., Seamans J.K. (2008). The dual-state theory of prefrontal cortex dopamine function with relevance to catechol-o-methyltransferase genotypes and schizophrenia. Biol. Psychiatry.

[25] Seamans J.K., Yang C.R. (2004). The principal features and mechanisms of dopamine modulation in the prefrontal cortex. Prog. Neurobiol..

[26] Rolls E.T., Loh M., Deco G. (2008). An attractor hypothesis of obsessive-compulsive disorder. Eur. J. Neurosci..

[27] Kapur S. (2003). Psychosis as a state of aberrant salience: A framework linking biology, phenomenology, and pharmacology in schizophrenia. Am. J. Psychiatry.

[28] Coyle J.T. (2012). NMDA receptor and schizophrenia: A brief history. Schizophr. Res..

[29] Krystal J.H., D’Souza D.C., Mathalon D., Perry E., Belger A., Hoffman R. (2003). NMDA receptor antagonist effects, cortical glutamatergic function, and schizophrenia: Toward a paradigm shift in medication development. Psychopharmacology.

[30] Bubeníková-Valesová V., Horácek J., Vrajová M., Höschl C. (2008). Models of schizophrenia in humans and animals based on inhibition of NMDA receptors. Neurosci. Biobehav. Rev..

[31] Coyle J.T., Tsai G., Goff D. (2003). Converging evidence of NMDA receptor hypofunction in the pathophysiology of schizophrenia. Ann. N. Y. Acad. Sci..

[32] Moghaddam B., Javitt D.C. (2012). From revolution to evolution: The glutamate hypothesis of schizophrenia and its implication for treatment. Neuropsychopharmacology.

[33] Kantrowitz J.T., Javitt D.C. (2010). N-methyl-d-aspartate (NMDA) receptor dysfunction or dysregulation: The final common pathway on the road to schizophrenia?. Brain Res. Bull..

[34] Millan M.J., Fone K., Steckler T., Horan W.P. (2014). Negative symptoms of schizophrenia: Clinical characteristics, pathophysiological substrates, experimental models and prospects for improved treatment. Eur. Neuropsychopharmacol..

[35] Strauss G.P., Cohen A.S. (2017). A Transdiagnostic Review of Negative Symptom Phenomenology and Etiology. Schizophr. Bull..

[36] Coley A.A., Gao W.J. (2018). PSD95: A synaptic protein implicated in schizophrenia or autism?. Prog. Neuropsychopharmacol. Biol. Psychiatry.

[37] Hahn C.G., Wang H.Y., Cho D.S., Talbot K., Gur R.E., Berrettini W.H., Bakshi K., Kamins J., Borgmann-Winter K.E., Siegel S.J. (2006). Altered neuregulin 1-erbB4 signaling contributes to NMDA receptor hypofunction in schizophrenia. Nat. Med..

[38] Panov G., Panova P. (2023). Obsessive-compulsive symptoms in patient with schizophrenia: The influence of disorganized symptoms, duration of schizophrenia, and drug resistance. Front. Psychiatry.

[39] Panov G. (2022). Dissociative Model in Patients with Resistant Schizophrenia. Front. Psychiatry.

[40] Panov G. (2022). Comparative Analysis of Lateral Preferences in Patients with Resistant Schizophrenia. Front. Psychiatry.

[41] Panov G. (2022). Gender-associated role in patients with schizophrenia. Is there a connection with the resistance?. Front. Psychiatry.

[42] Panov G. (2022). Higher Depression Scores in Patients with Drug-Resistant Schizophrenia. J. Integr. Neurosci..

[43] Panov G.P. (2022). Early Markers in Resistant Schizophrenia: Effect of the First Antipsychotic Drug. Diagnostics.

[44] Panov G., Djulgerova S., Panova P. (2022). The effect of education level and sex differences on resistance to treatment in patients with schizophrenia. Bulg. Med..

[45] Panov G., Djulgerova S., Panova P. (2022). Comparative anthropometric criteria in patients with resistant schizophrenia. Bulg. Med..

[46] Panov G., Djulgerova S., Panova P., Stefanova S. (2024). Untangling Depression in Schizophrenia: The Role of Disorganized and Obsessive-Compulsive Symptoms and the Duration of Untreated Psychosis. Biomedicines.

[47] Panov G., Panova P., Dyulgerova S., Chakarov I. (2026). From Signal to Symptom: EEG Paroxysms and Background Slowing as Potential Biomarkers and Compensatory Failures in Treatment-Resistant Schizophrenia. Biomedicines.

[48] Potter M.C., Elmer G.I., Bergeron R., Albuquerque E.X., Guidetti P., Wu H.Q., Schwarcz R. (2010). Reduction of endogenous kynurenic acid formation enhances extracellular glutamate, hippocampal plasticity, and cognitive behavior. Neuropsychopharmacology.

[49] Han Q., Cai T., Flavavin M.A., Li J. (2010). Structure, expression, and function of kynurenine aminotransferases in human and rodent brains. Cell. Mol. Life Sci..

[50] Erhardt S., Schwieler L., Imbeault S., Engberg G. (2017). The kynurenine pathway in schizophrenia and bipolar disorder. Neuropharmacology.

[51] Wonodi I., Schwarcz R. (2010). Cortical kynurenine pathway metabolism: A novel target for cognitive enhancement in schizophrenia. Schizophr. Bull..

[52] Linderholm K.R., Skogh E., Olsson S.K., Dahl M.L., Holtze M., Engberg G., Samuelsson M., Erhardt S. (2012). Increased levels of kynurenine and kynurenic acid in the CSF of patients with schizophrenia. Schizophr. Bull..

[53] Rassoulpour A., Wu H.Q., Ferre S., Schwarcz R. (2005). Nanomolar concentrations of kynurenic acid reduce extracellular dopamine levels in the striatum. J. Neurochem..

[54] Vécsei L., Szalárdy L., Fülöp F., Toldi J. (2013). Kynurenines in the CNS: Recent advances and new questions. Nat. Rev. Drug Discov..

[55] Müller N., Weidinger E., Leitner B., Schwarz M.J. (2015). The role of inflammation in schizophrenia. Front. Neurosci..

[56] Alkondon M., Pereira E.F.R., Albuquerque E.X. (2011). Endogenous activation of nAChRs and NMDA receptors contributes to the excitability of CA1 stratum radiatum interneurons in rat hippocampal slices: Effects of kynurenic acid. Biochem. Pharmacol..

[57] Lisman J.E., Coyle J.T., Green R.W., Javitt D.C., Benes F.M., Heckers S., Grace A.A. (2008). Circuit-based framework for understanding neurotransmitter and risk gene interactions in schizophrenia. Trends Neurosci..

[58] Uhlhaas P.J., Singer W. (2010). Abnormal neural oscillations and synchrony in schizophrenia. Nat. Rev. Neurosci..

[59] Yang Q., Zhang Y., Yang K., Niu Y., Fan F., Chen S., Luo X., Tan S., Wang Z., Tong J. (2022). Associations of the serum kynurenine pathway metabolites with P50 auditory gating in non-smoking patients with first-episode schizophrenia. Front. Psychiatry.

[60] Martin L.F., Freedman R. (2007). Schizophrenia and the alpha7 nicotinic acetylcholine receptor. Int. Rev. Neurobiol..

[61] Thuné H., Recasens M., Uhlhaas P.J. (2016). The 40-Hz Auditory Steady-State Response in Patients With Schizophrenia: A Meta-analysis. JAMA Psychiatry.

[62] Khater S.I., Lotfy M.M., Alandiyjany M.N., Alqahtani L.S., Zaglool A.W., Althobaiti F., Ismail T.A., Soliman M.M., Saad S., Ibrahim D. (2022). Therapeutic Potential of Quercetin Loaded Nanoparticles: Novel Insights in Alleviating Colitis in an Experimental DSS Induced Colitis Model. Biomedicines.

[63] Olsson S.K., Andersson A.S., Linderholm K.R., Holtze M., Nilsson-Todd L.K., Schwieler L., Olsson E., Larsson K., Engberg G., Erhardt S. (2009). Elevated levels of kynurenic acid change the dopaminergic response to amphetamine: Implications for schizophrenia. Int. J. Neuropsychopharmacol..

[64] Klimesch W. (1999). EEG alpha and theta oscillations reflect cognitive and memory performance: A review and analysis. Brain Res. Rev..

[65] Klimesch W. (2012). Alpha-band oscillations, attention, and controlled access to stored information. Trends Cogn. Sci..

[66] Singer W. (2013). Cortical dynamics revisited. Trends Cogn. Sci..

[67] Jensen O., Mazaheri A. (2010). Shaping functional architecture by oscillatory alpha activity: Gating by inhibition. Front. Hum. Neurosci..

[68] Arnal L.H., Giraud A.L. (2012). Cortical oscillations and sensory predictions. Trends Cogn. Sci..

[69] Foxe J.J., Snyder A.C. (2011). The role of alpha-band brain oscillations as a sensory suppression mechanism during selective attention. Front. Psychol..

[70] Bazanova O.M., Vernon D. (2014). Interpreting EEG alpha activity. Neurosci. Biobehav. Rev..

[71] Kronbichler L., Tschernegg M., Martin A.I., Schurz M., Kronbichler M. (2017). Abnormal Brain Activation During Theory of Mind Tasks in Schizophrenia: A Meta-Analysis. Schizophr. Bull..

[72] Kane J.M., Agid O., Baldwin M.L., Howes O., Lindenmayer J.P., Marder S., Olfson M., Potkin S.G., Correll C.U. (2019). Clinical Guidance on the Identification and Management of Treatment-Resistant Schizophrenia. J. Clin. Psychiatry.

[73] Lally J., Gaughran F., Timms P., Curran S.R. (2016). Treatment-resistant schizophrenia: Current insights on the pharmacogenomics of antipsychotics. Pharmgenom. Pers. Med..

[74] Barkmeier D.T., Loeb J.A. (2009). An animal model to study the clinical significance of interictal spiking. Clin. EEG Neurosci..

[75] Newson J.J., Thiagarajan T.C. (2019). EEG Frequency Bands in Psychiatric Disorders: A Review of Resting State Studies. Front. Hum. Neurosci..

[76] Boutros N.N., Arfken C., Galderisi S., Warrick J., Pratt G., Iacono W. (2008). The status of spectral EEG abnormality as a diagnostic test for schizophrenia. Schizophr. Res..

[77] De Pieri M., Rochas V., Sabe M., Michel C., Kaiser S. (2024). Pharmaco-EEG of antipsychotics’ response: A systematic review. Eur. Psychiatry.

[78] Gordillo D., da Cruz J.R., Chkonia E., Lin W.H., Favrod O., Brand A., Figueiredo P., Roinishvili M., Herzog M.H. (2023). The EEG multiverse of schizophrenia. Cereb. Cortex.

[79] American Psychiatric Association (2013). Diagnostic and Statistical Manual of Mental Disorders.

[80] World Health Organization (1992). The ICD-10 Classification of Mental and Behavioural Disorders: Clinical Descriptions and Diagnostic Guidelines.

[81] Kane J., Honigfeld G., Singer J., Meltzer H. (1988). Clozapine for the treatment-resistant schizophrenic. A double-blind comparison with chlorpromazine. Arch. Gen. Psychiatry.

[82] Andreasen N.C., Carpenter W.T., Kane J.M., Lasser R.A., Marder S.R., Weinberger D.R. (2005). Remission in schizophrenia: Proposed criteria and rationale for consensus. Am. J. Psychiatry.

[83] Kay S.R., Fiszbein A., Opler L.A. (1987). The Positive and Negative Syndrome Scale (PANSS) for schizophrenia. Schizophr. Bull..

[84] Overall J.E., Gorham D.R. (1962). The Brief Psychiatric Rating Scale. Psychol. Rep..

[85] Kane N., Acharya J., Benickzy S., Caboclo L., Finnigan S., Kaplan P.W., Shibasaki H., Pressler R., van Putten M.J.A.M. (2017). A revised glossary of terms most commonly used by clinical electroencephalographers and updated proposal for the report format of the EEG findings. Revision 2017. Clin. Neurophysiol. Pract..

[86] Noachtar S., Remi J. (2009). The role of EEG in epilepsy: A critical review. Epilepsy Behav..

[87] Makeig S., Delorme A., Westerfield M., Jung T.P., Townsend J., Courchesne E., Sejnowski T.J. (2004). Electroencephalographic brain dynamics following manually responded visual targets. PLoS Biol..

[88] Nunez P.L., Srinivasan R. (2006). Electric Fields of the Brain: The Neurophysics of EEG.

[89] Delorme A., Makeig S. (2004). EEGLAB: An open source toolbox for analysis of single-trial EEG dynamics including independent component analysis. J. Neurosci. Methods.

[90] Duffy F.H., Hughes J.R., Miranda F., Bernad P., Cook P. (1994). Status of quantitative EEG (QEEG) in clinical practice, 1994. Clin. Electroencephalogr..

[91] Janssens S.E.W., Sack A.T., Ten Oever S., Graaf T.A. (2022). Calibrating rhythmic stimulation parameters to individual electroencephalography markers: The consistency of individual alpha frequency in practical lab settings. Eur. J. Neurosci..

[92] Koshiyama D., Miyakoshi M., Tanaka-Koshiyama K., Joshi Y.B., Sprock J., Braff D.L., Light G.A. (2021). Abnormal phase discontinuity of alpha- and theta-frequency oscillations in schizophrenia. Schizophr. Res..

[93] Olejarczyk E., Bogucki P., Sobieszek A. (2017). The EEG split alpha peak: Phenomenological origins and methodological aspects of detection and evaluation. Front. Neurosci..

[94] Klimesch W. (1997). EEG-alpha rhythms and memory processes. Int. J. Psychophysiol..

[95] Field A. (2018). Discovering Statistics Using IBM SPSS Statistics.

[96] JASP Team (2024). JASP.

[97] Panov G., Stoyanov D., Draganski B. (2023). Quantitative EEG in patients with schizophrenia. Computational Neuroscience.

[98] Panov G., Panova P. (2024). Neurobiochemical Disturbances in Psychosis and their Implications for Therapeutic Intervention. Curr. Top. Med. Chem..

[99] Garrett D.D., Samanez-Larkin G.R., MacDonald S.W., Lindenberger U., McIntosh A.R., Grady C.L. (2013). Moment-to-moment brain signal variability: A next frontier in human brain mapping?. Neurosci. Biobehav. Rev..

[100] Blaauboer A., van Koetsveld P.M., Mustafa D.A.M., Dumas J., Dogan F., van Zwienen S., van Eijck C.H.J., Hofland L.J. (2022). The Class I HDAC Inhibitor Valproic Acid Strongly Potentiates Gemcitabine Efficacy in Pancreatic Cancer by Immune System Activation. Biomedicines.

[101] Turrini S., Bevacqua N., Cataneo A., Chiappini E., Fiori F., Battaglia S., Romei V., Avenanti A. (2023). Neurophysiological Markers of Premotor–Motor Network Plasticity Predict Motor Performance in Young and Older Adults. Biomedicines.

[102] Rajkumar R.P. (2023). Biomarkers of Neurodegeneration in Post-Traumatic Stress Disorder: An Integrative Review. Biomedicines.

